# The Plastic Glial-Synaptic Dynamics within the Neuropil: A Self-Organizing System Composed of Polyelectrolytes in Phase Transition

**DOI:** 10.1155/2016/7192427

**Published:** 2016-02-01

**Authors:** Vera Maura Fernandes de Lima, Alfredo Pereira

**Affiliations:** ^1^Centro de Biotecnologia, IPEN-CNEN/SP, Avenida Prof. Lineu Prestes 2242, Butantã, 05508-000 São Paulo, SP, Brazil; ^2^Institute of Biosciences of Botucatu, São Paulo State University (UNESP), Campus Rubião Jr., 18618-970 Botucatu, SP, Brazil

## Abstract

Several explanations have been proposed to account for the mechanisms of neuroglial interactions involved in neural plasticity. We review experimental results addressing plastic nonlinear interactions between glial membranes and synaptic terminals. These results indicate the necessity of elaborating on a model based on the dynamics of hydroionic waves within the neuropil. These waves have been detected in a small scale experimental model of the central nervous system, the* in vitro* retina. We suggest that the brain, as the heart and kidney, is a system for which the state of water is functional. The use of nonlinear thermodynamics supports experiments at convenient biological spatiotemporal scales, while an understanding of the properties of ions and their interactions with water requires explanations based on quantum theories. In our approach, neural plasticity is seen as part of a larger process that encompasses higher brain functions; in this regard, hydroionic waves within the neuropil are considered to carry both physiological and cognitive functions.

## 1. Introduction

We discuss the plastic nonlinear interactions between glial membranes and synaptic terminals, using physical-chemical (electrochemical) concepts to describe the basic brain dynamics that carry psychophysical processes. In our approach, the Hodgkin-Huxley membrane model was not relevant for the explanation of experimental results. Instead, Ichigi Tasaki's membrane model [1–4] and Katchalsky's [5–7] proposed synapse model are the foundations of the interpretation of our electrophysiological and optical data obtained from experiments with* in vitro* retinas.

The spreading depression is a general central grey matter phenomenon predicted by Lashley in 1941 [8]. There is an aphorism in this field of research that “to understand spreading depression is to understand the brain.” Based on the evolution of his own scotomas preceding a full-fledged migraine attack, Lashley predicted an excitation/inhibition wave spreading in the primary visual cortex and correctly estimated its velocity at 3 mm/min. The distortion of perception that accompanies the wave made him interpret it as a psychophysical phenomenon, as expressed in the paper's title [8].

This model of brain activity advanced by Lashley has not been hegemonic within the neurosciences community but was kept alive by a minority of researchers. For example, just two years after Tasaki and Chang's paper on cortical glial cells slow potentials [9] and its implicit contribution to the EEG, Galambos [10] published an enthusiastic review of the role of glia in the brain. The role of polyelectrolytes (in this case polyanions) in synapses and their role in the brain self-organization were clearly stated by Katchalsky [6, 7] in the late sixties and early seventies of the XX century.

The explanatory value of a scientific conjecture can be confirmed if it permits clear predictions and these predictions are supported by experimental results. We quote directly Neumann and Katchalsky [11]: “Controlled changes in the environment of metastable macromols or subcellular macromol. organizations such as membranes by high elec. fields or by ion gradients can induce conformational changes which could serve as reproducible imprints of a memory nature.” Note that the supramolecular structure of biological polyanionic gels creates electromagnetic fields that in turn influence the metastable state of these gels (a case of “circular causation,” a concept that plays a central role in synergetics and systems control theories). Estimations of these fields range in the order of 300,000 V/cm^2^ [6]. Shortly after this prediction, in 1977, a type of long-term memory (with a duration of two weeks) was demonstrated in the basement membrane of striated muscles [12]. This finding has been confirmed many times over (see, e.g., [13–15]). These results led to the suggestion that information could be embodied not in special molecules, but in the energy pattern generated by the macroscopic supramolecular structure.

A second vantage of the electrochemical approach to the neuropil is that it is a unifying explanation for apparently disparate research results: for instance, the effects of lithium on the heart and its usage in psychiatry to control bipolar disorder; the effects of cationic proteins in both tissues; the physical-chemical (i.e., the solvent and not the isotope) effects of deuterium in the retina model, as well as in psychiatry (explaining the reported increase of depression in populations submitted to high concentration of deuterium in drinking water; see [16]).

All the above results are discussed in the following sections. We begin with deuterium as solvent in two electrochemical systems; then, we discuss the role of the basement membrane in the emergence of dynamic structures and proceed to discuss the limitations of electrophysiological recordings within the neuropil, when these dynamic patterns are studied. The presence of remarkable intrinsic optic signals (IOS) in retinal grey matter adds a new dimension to these studies. Two recent publications give a detailed description of the retinal IOS and how to use it in research [17, 18].

## 2. The Deuterium Experimental Results and the Fourth State of Water

The deuterium solvent experiments series complemented a long series of experiments aimed at comparing the behavior of two very convenient excitable media systems: the excitation states in* in vitro* retinas and the Belousov-Zhabotinsky reaction system (B-Z; for the definition and history of this chemical system, see Zhabotinsky's article in Scholarpedia [19]). Both are well-known excitable media and share the same descriptive mathematical dynamics. Zykov [20] states the mathematical identity between the biological equations of FitzHug Nagumo and Brusselator [21] of Ilya Prigogine chemical equations systems. In addition, there is a very good definition of excitable media in the chapter.

We have analyzed dynamical waves in retinas as a two-dimensional electrochemical pattern. In Brazil, the first set of comparative experiments in biological and chemical system was made in 1980: a circling B-Z system in analogy with the circling retinal waves created by Martins-Ferreira in 1974 [22, 23]. Later on, one of the authors was part of a research group that proceeded to compare the two systems' behavior using modulation of physical fields, electromagnetic and gravitational [24–26]. The similarity in response was uncanny. For example, at similar temperature, the circling retinal waves and B-Z waves spread at very close velocities, and the retina and the B-Z system in gel react to increases of gravitational forces and to microgravity in the same qualitative way.

Deuterium solutions have different physical properties than water solutions, as shown by Katz in 1960 [27]. The use of liquid deuterium as solvent interferes with the global coupling observed in excitable media [28, 29]. Liquid deuterium and water have similar dielectric constant and surface tension. By contrast, the viscosity of deuterium at 25°C is 25% greater than that of water. Also significant is the temperature of maximum density (g/cc): 3.98°C for water and 11.2°C for deuterium. This difference made researchers speculate that some effects of liquid deuterium would mimic temperature effects [27, 30, 31]. However, the greater difference (except for mass) between the isotopes is in self-ionization: liquid deuterium dissociation is five times smaller than that of water.

Previous experimental research with deuterium gave a 20% lower flow within aquaporins [32] for deuterium compared to water. Based on earlier reports that the deuterium effect was equal to a temperature difference of 4 degrees [30, 31], we expected a milder effect on retinas and a larger effect on the B-Z reaction system. Zhabotinsky remarked that “the* Belousov-Zhabotinsky (BZ) reaction* is a family of oscillating chemical reactions. During these reactions, transition-metal ions catalyze oxidation of various, usually organic, reductants by bromic acid in acidic water solution…. The BZ reaction can generate up to several thousand oscillatory cycles in a closed system, which permits studying chemical waves and patterns without constant replenishment of reactants” [19].

In spite of the expectation, retinal excitation waves and B-Z systems responded in the same way, with complete collapse of excitability in a short time. This was a very powerful change in both systems. The role of self-ionization of water in spreading depression was unclear until we learned about the work of Pollack and Bunkin with interfacial water close to polyelectrolytes [33–38]. To summarize these results, we can say that when water is close to hydrophilic polyanions (or cations), it assumes a state that has the properties of* quasi*-liquid crystals! [39]. For example, the Bunkin group found that its refractive index is 1.46 (while water has 1.332) and that the interface is anisotropically sharing the optical properties of photonic crystals. This structure is very dynamic and fragile and disrupted by ultrasound. Therefore, when liquid deuterium substitutes water, the diminished capacity for self-ionization impairs the formation and increases the fragility of these crystals and the system excitability collapses.

**Figure 1:**
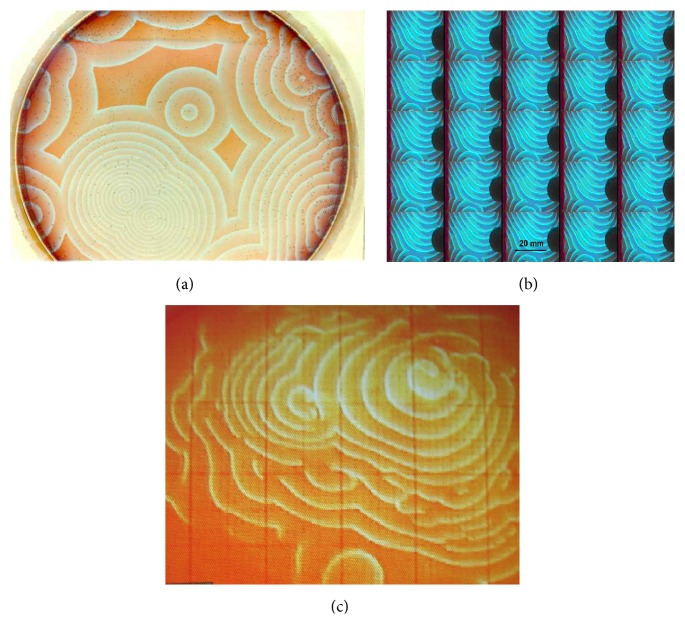
Experiment with the B-Z system. Clockwise: (a) the catalyzer ferroin was fixed in silica gel. One hour after covering the gel with the reactants, several centers begin to create propagating circular waves that cause annihilation of each other upon collision. The whole Petri dish (10 cm diameter) is filled with the self-organized spatiotemporal pattern; (b) detail of another experiment showing the temporal evolution of the B-Z system using water as solvent; (c) experiment of B-Z in gel in which deuterium was used as solvent. Only about one-third of the Petri dish is occupied by the propagating waves. The grid overlaying the dish has 2.0 cm spacing. A few reactive centers send waves that died out long before reaching the border of the Petri dish. The waves become “fuzzy” and disappear. The propagation velocity was five times slower than the ones in the water gel. The deuterium systems also were short-lived compared to the water systems. The results suggest the importance of dissociated water in the B-Z system (figure modified from [28]).

A very important point to be made for the structured interfacial water is that it stores electrical energy and a negative potential surges at the interface. At the interface with the synthetic Nafion, this structure has a range of more than 100 *µ*m and a negative potential of 120 mV. This negative interfacial potential can explain the three-to-four mV potential drop one measures in electrophysiological recordings when one electrode enters the tissue. In Figures 1(a) and 1(c), we show two self-organized wave patterns of B-Z reaction in gel using water (a) and deuterium as solvent (c). The spatial scale of the pattern shown in (c) is four times amplified compared to (a).

In water, B-Z waves occupy the whole Petri dish within the first hour of the experiment. In deuterium, only a fraction of the dish showed waves and these waves had blurred endings and died before travelling to the border.

Out of seven retinas submitted to liquid deuterium, two (from the same animal) were dead within the hour. Both displayed the typical excitotoxic optical profile before tissue death (see [18]). The other five became unstable and developed “spontaneous” activity [29], in which the optical profiles also changed from wave to wave. Some of these optical profiles had resemblance to waves recorded at 20°C [40], whereas others had the absence of the second peak one finds in low glucose conditions leading to tissue exhaustion [41]. After one hour or so of such activity, the retinas were unexcitable for the next two hours of washing off the deuterium, when the experiments were finished.

Experimental results at the extracellular matrix of the inner plexiform layer of chick retinas showed a fast (in the order of seconds) pH shift in the alkaline direction at the wave onset [42]. Similar records made intracellularly in astrocytes [43] showed a similar pH shift during spreading depression. It is tempting to interpret this early alkaline shift as the loss of the self-ionized hydronium ions around the polyanions. The Glial Fibrillary Acidic Protein appears to be the ideal candidate for inducing the formation of* quasi*-liquid crystals inside the cells. Thus, the depolarization of spreading depression could well be dominated by the transient loss of this structure intra- and extracellularly and therefore the loss of transparency.

We learned from the liquid deuterium experiments the crucial role of hydronium ions in the brain. The next important question was as follows: what is the brain doing with the energy of the interfacial water?

## 3. The Basement Membrane/Glial Endfeet CNS Interface and Its Interactions with Complex Molecules

The experiments presented in this section have in common complex molecules that did not easily penetrate the tissue and hence had to interact with the basement membrane glial endfeet. They showed a powerful transducer role for the tissue interface.

The glycoside ouabain is a large hydrophilic molecule and as such it is poorly permeable in lipid membranes. On the other hand, it is a well-known blocker of the Na/K ATPase activity. The putative place of the blockade is an extracellular loop close to the lipid bilayer. Therefore, in order to reach the loop, ouabain has to negotiate a pathway through the 200 *µ*m thick layer of polyanions continuous to the lipid bilayer. We applied ouabain to* in vitro* chick retinas, first in circling experiments and later with exogenous pulses or slow perfusion [44].

In this avascular retina, the metabolism depends one hundred percent on anaerobic glycolysis performed in the glia [45]. Moreover, there is tight coupling between the rate of ion transport and the production of lactate by glia (for details and relevant literature, see [18, 45]). The consequence of stopping ion transport is a halt in metabolism and subsequent change in the ATP/ADP ratio. We used short pulses as well as slow perfusion to apply ouabain from 1 mM to 10 nM concentration [44]. All 10 retinas were dead within one to two hours of exposition to ouabain. In this series of experiments, we could distinguish two pathways to tissue death: an acute cell lysis that has the appearance of a macroscopic edema [18, 45] and a second type of evolution to death (apoptosis?) without the edema.

At the lowest concentration of 10 nM, the onset of the excitotoxic response had a latency of 30 minutes whereas the onset was 20–30 seconds with 1 mM. The nanomolar concentration range is the hormonal range and indeed the hypothalamus and suprarenal glands produce cardiac glycosides (see references at [18, 44]). It happens that the *β* subunit of the glia ATPase is an adhesion molecule [46, 47]. In epithelium, adhesion molecules are closely linked to apoptosis. Therefore, the tissue death induced by nanomolar concentration of ouabain could be a consequence of subunit interaction mechanisms rather than ion transport blockade. It was in the lowest concentration that we observed slow death without the macroscopic edema of acute cell lysis. All in all, these experiments showed a dominant role of the glial network in the control of the tissue state.

The ubiquitous Na/K ATPase exemplified the transducer role of the glia. Phase transitions also have an important role in the spread of excitation. Gyroxin (28 KD) is a toxin of the Brazilian rattlesnake venom (2% of the proteins), which caused seizures in rats and mice, but did not have explanatory mechanisms for its CNS action until we applied it to* in vitro* retinas [48]. It has enzymatic activity as a serinoprotease like the thrombin, and up to 30% of its weight is carbohydrate. It is one unlikely candidate to cross the blood brain barrier and yet it produces seizures in rats and mice.

At high concentration short pulses (0.500 *µ*g/500 *µ*L), the rise of the excitotoxic response was so fast that it precluded the toxin entering the tissue. The change had to be at the basement membrane endfeet interface. The change in optical properties was five to six times larger than that measured at the peak of the first optical component of the retinal spreading depression waves. The change in the optical signal was in the millisecond range. For comparison, a similar large pulse of glutamate (1 mM/500 *µ*L) elicits excitotoxicity with a latency of 4 seconds. With the large volume pulse, the IOS of the whole central retina changed at once. In order to see propagation of this change, we used micro pulses with only 50 *µ*L volume aspersed close to the retina surface. In this case, we could see propagation of the excitation with speed also up to five times the spread of retinal circular waves.

Serinoproteases like thrombin cause platelet aggregation and this aggregation is in itself an abrupt change akin to phase transitions. Essays made to compare gyroxin and thrombin produced similar activity for both [48]. This action is due to PAR (Protein Activated Receptors members of the 7TM receptor family). These receptors have a prominent extracellular loop that is cut by the activating enzyme in an irreversible faction. That apparently was the trigger for the gyroxin excitotoxic response.* A conformational change that spreads through the basement membrane* is a clear example of phase transition in polyelectrolytes that abruptly changes the glia neural dynamics. In mammalian CNS, PAR were localized in glia membrane and endothelial basement membrane. Glia PAR activation leads to neuronal activation [49]. The retinal gyroxin results confirmed the interpretation of neural glial dynamics in slices. Because chicken retina is avascular, no vascular component of the basement membrane was present at the vitreal retina interface; only the glia basement membrane contacted the gyroxin. To explain the seizures in rats and mice, either gyroxin acted at the AV3V system in which the blood brain barrier does not exist or gyroxin could cause breakdown of the barrier. Essays confirmed that it did cause this breakdown [48]. This mechanism of triggering seizures could only be proposed due to the added dimension of the strong intrinsic optical signal present in retinal experiments.

The membrane model that assigns a prominent role for phase transition in polyelectrolytes is the Tasaki model [2, 3]. The phase transitions of interest are “volume phase transitions” of polyelectrolytes. A change in electrical field or of ion activity can bring about abrupt changes in polyanions. Once started, they spread through the network. Within this model, the action potential is an electrochemical wave propagating at one dimension, while a retinal spreading depression wave is a wave in two dimensions, and a heartbeat is a propagating pattern in three dimensions. At least for the* in vitro* retina, it is long known that glia and extracellular matrix are sufficient to maintain propagation of a wave without changes in the optical signal and at the same velocity of the neuropil spread. This is due to the optical nerve papilla, a place where only exiting axons and glia are present. It covers a relatively large area continuous to the pecten. Waves invade this area without changes in the optical signal. The Müller cells of chicken retina also do not have gap junctions and have only very few mitochondria. These are located close to the receptor layer far away from the inner plexiform layer, the place where all wave concomitants are at maximum value [17, 18]. Thus, theories assigning important roles for gap junctions or mitochondria in spreading depression do not agree with results of retina experiments. The propagation within the papilla shows that the glial network is necessary and sufficient for maintaining propagation, while the gyroxin experiments showed that the endfeet layer can initiate excitotoxic responses that propagate in the system.

In the previous section, we described a pathway toward tissue excitability collapse in the presence of deuterium. The tissue lost transparency and hence functionality. In here, we describe another pathway toward excitability collapse in which transparency increased and the tissue appeared in very good shape. We refer to the experiments with protamine, a small (32 amino acids), cationic, naturally occurring protein. In medicine, the avidity of protamine for heparan sulfate (heparin) is used in patients being weaned off anticoagulant treatment. The protein is a polyarginine that in acid pH can acquire a charge of +11. Heparan sulfate is a constituent of glycocalyx and basement membranes (indeed, it is very hard to decide when glycocalyx ends and the basement membrane begins). Essays* in vitro* [50] with heparinized human plasma showed that protamine made stable (up to 4 hours), large (100 nm) heteropolymers complexes with heparin in plasma.

In retinas, the results were just fascinating [51]: protamine induced excitability collapse in 32–34 minutes at concentrations of 0.625 and 1.25 nM [51]. Several waves were observed in this interval. A video recorded the passage of waves in central retina, and optical profiles were acquired at two spatial dimensions, a macro profile obtained from a photomultiplier sampling a circular area of 1 mm diameter and a 50 *µ*m square matrix of pixels obtained from a camera signal. This micro optical signal recorded as close as possible to an electrode tip that measured the potential drop associated with the retinal waves. The photomultiplier counted the amount of scattered light by the retina and its baseline quantified the shifts in transparency. The retinas just became more and more transparent with time, and the observed waves became split with larger and larger areas not invaded by the waves. With 22.7 nM, the excitability collapse occurred in three minutes. More interestingly, the wave optical profiles just became smaller such that if one compares the profile obtained before the protamine application with the last obtained before complete collapse of excitability, the later looks like a miniature version of the first, suggesting that membrane mechanisms and tissue metabolism were not affected by the protein. It appeared that the heteropolymers created by the cationic peptide were far away from the endfeet membrane and the effect was purely physical, a type of mechanical clamping of the basement membrane that prevents wave propagation.

Two facts support this interpretation: first, a functional retina is a transparent one and the degree of transparency is extremely sensitive to membrane states and tissue metabolic state; second, mechanical stimuli did irritate the tissue and with strong mechanical touch that can create large lesions, we could see initial propagation that died out after 250–300 *µ*m. With these results, we could predict a similar effect on heart and axons. Indeed, protamine blocks action potentials in axons [51]. In the heart, we found a report of blockade of a* transplanted* human heart by protamine. This precluded neural mechanisms and the effect could only be intrinsic [52].* We see the same effect of basement membranes on the propagation of electrochemical patterns at one, two, and three spatial dimensions of biological systems*, in all cases caused by the same agent. At vessels and epithelium, respectively, protamine blocked the rolling over of leucocytes and was found to alter the structure of the epithelial basement membrane while not affecting gap junctions [53, 54]. Neither had any effect on the membrane apical potential.

Another cationic protein applied to retinas was crotamine [51]. It is also small (42 amino acids in the primary chain of which 9 are lysines and 2 arginines). In acid pH, it acquires a charge of +9. It is also a Brazilian rattlesnake toxin that belongs to a class of small basic toxins. Its primary chain is identical to the* Naja naja* P6 toxin. Like gyroxin, it produces a complex sequence of stereotyped behavior in mice (for details, see [51]).

With 9 lysines in the primary chain, crotamine is a naturally occurring polylysine. Polylysines adsorb on red blood cells membranes and red cell “ghosts” [6]. They raise the surface potential of the cells and at a critical potential promote agglutination of the cells or membranes “ghosts.” This critical potential lies well below the maximum potential. The critical potential measured with potentiometers was ~−10 mV and with increasing adsorption of the peptide surface potential reached more than +20 mV [6].

We applied crotamine with short pulses of 500 *µ*g/500 *µ*L (206 nM) with pipettes. The perfusion was stopped for a short time and then resumed. The baseline transparency shifted with a latency too short to be measurable and reached a plateau after 5 to 6 minutes. Waves elicited after 12 minutes of crotamine exposure had an altered profile with a small first optical peak, slow recovery, and a high amplitude long duration second peak. The altered parameters of the profiles indicate that all membrane mechanisms, from the dissipation of the electrochemical gradients at wave onset and its recovery, were disturbed. Upon resumption of perfusion, the transparency baseline recovered and the profiles reverted to the typical pattern in 15 minutes, suggesting an electrostatic effect and a weak interaction with the endfeet membrane. The wave profile pattern recorded in the presence of crotamine suggested modulation of the rate of the Na/K ATPase ion transfer.

**Figure 2:**
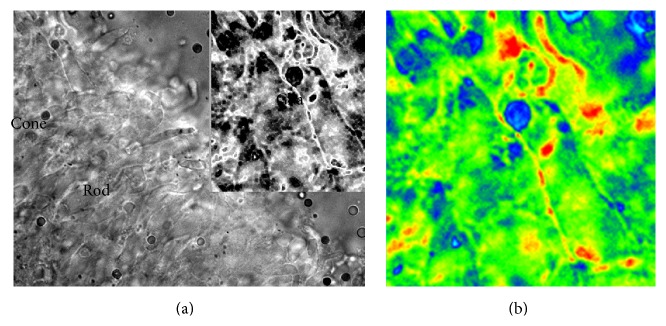
Fluorescence in Müller cell. (a) Fresh thin slice of retina under UV illumination. The slice was previously incubated with fluorescent crotamine [51]. The outer retina receptor layer has strong fluorescence. Part of the fluorescence is intrinsic, due to oil droplets (blue, green, and red) found in the inner segment of cones. A rod outer segment and a cone inner segment can easily be seen. Close to the cone, the inner segment microvilli of the outer retina portion are very bright. (b) False color display of the inset. Note that all the fluorescence in glia is extrinsic or due to the close association (adsorption) of the basic protein with glial membrane.

In Figure 2, we show the photomicrograph under UV illumination of characteristic microvilli of the outer segment of an intensely labelled Müller cell. Fresh retinal slices incubated with fluorescent crotamine (see [51]) under UV illumination showed intense fluorescence associated with the glial membrane, compared with the inner segment of the adjacent cone receptor cell that also shows some fluorescence. In these experiments, one could see bright spots inside the Müller cells indicating fast internalization of the protein by glia. It appears that the adsorption of crotamine induces endocytosis of membrane patches. In additional experiments, we showed that crotamine is easily incorporated into lipid monolayers and bilayers and that the protein itself forms oligomers in aqueous solution due to its amphiphilic nature [55].

The crotamine effect on muscle cells is depolarization and cell death. The place of crotamine action was first supposed to be sodium channels, while the cell death effect of a similar basic toxin, the P6 toxin, with the same 9 lysines and 2 arginines in the primary chain, was attributed to the Na/K ATPase [56–59]. More recently, research in molecular biology did not confirm sodium channel interaction with crotamine [60]. Why would the adsorption of crotamine kill muscle and spare retinas? The pump subunits assembled in muscle and glia are different enough to change their physiology: muscle pump is driven by sodium inside cells, whereas glia pump is driven by potassium outside [61]. Not only the distribution of *α* subunits but also their associated *β* subunits (the glycosylated subunits) differ in CNS and heart. Irrespective of the subunits expressed, all of the membrane ATPases have several functions besides ion transport, including the apoptotic and acute cell lysis pathways [62].

Ouabain promotes acute cell lysis and apoptosis in the central gray matter of cortex [63] and retina [44], and in muscle it modulates ion transport without killing the tissue. While crotamine modulates ion transport in central gray matter and depolarizes the glial network without further harm, it kills muscle and tumors cells either with acute cell lysis or with apoptosis [56, 57]. The depolarization of the glial network in mice initiates stereotyped behavior that ends in a frozen characteristic posture followed by death [51].

If the results described by Katchalsky [6] for polylysines on red blood cells membranes are taken into account, then the crotamine effects are not classical lock-and-key effects on specific receptors, but a physical change in the membrane electrical field that changes the state of intrinsic and associated membrane proteins. The glial membrane network depolarization changes the tissue dynamics expressed in the tissue baseline transparency and parameters of the optical profiles of propagating waves.

The ouabain experiments merely reproduced in the retina the cortical effects of the glycoside [63], just showing one more time that the inner retina is part of the CNS and not just a model of it. The experiments reinforced the transducer role of the Na/K ATPase of the endfeet membrane. With gyroxin, we learned how fast the change in neural and glial dynamics can happen and spread after a trigger that promoted conformational changes in the basement membrane. The appropriate name for this is* phase transition*. Protamine and crotamine experiments suggest the adaptability of polyelectrolytes to the surrounding medium. If one thinks in very simple terms of stick-and-ball configuration, both proteins that are thought to be compact (disulfide bonds) can unfold under the influence of the electrical fields at the membrane interface. The stick configuration of the amphiphilic crotamine inserts itself in the membrane the same way that it does in bilayers and monolayers, while the more hydrophilic protamine repelled by the hydronium ions at the interface and in the basement membrane finds heparan sulfates and then makes stable heteropolymers that clamp pieces of the basement membrane, thus impairing polyelectrolytes conformational changes crucial for spread of excitation.

Last but not least, the remarkable IOS of retinas permit the observation not only of the velocity of spread of excitation, but also of the manner of spread. Excitotoxic responses spread very differently from circular waves. Also, we observed that there are several ways of tissue death. Acute cell lysis is observed while it is happening in retinas, instead of hours to days later which is the rule in cortex [44, 48, 63, 64]. The cause is the macroscopic tissue edema associated with acute cell lysis while the apoptotic pathway produces final states without the edema [44, 48, 62, 63].

## 4. The Potassium and Calcium Activities within the Neuropil during Spread of Excitation and within Dynamic Electrochemical Patterns

In this section, we apply the model to the neuropil, approaching the mechanisms involved in neural plasticity. We begin by describing results of measured activity with ion-sensitive electrodes inserted in the neuropil and the related extrinsic fluorescence. The layered structure of vertebrate retinas, in which cell bodies and synaptic terminals are completely segregated, gives rise to sharp potential profiles that can be better identified than in the hippocampus cell bodies'* stratum radiatum*. In the inner plexiform layer, there are only two types of membrane: glial and synaptic terminals. The microglia of this avascular retina is situated at the border of the ganglion cell layer and inner plexiform layer [45].

The ion activities changes during excitation waves are related to lyotropic effects of ions (or the Hofmeister series). The first researchers to do a systematic investigation of the lyotropic effect of ions on neural excitability were Tasaki et al. in 1965 [65]. A classical study in the field of stereochemical properties of ions is the review by Williams in 1970 [66]; more recent ones are by Collins in 2004 [67] and Zhang and Cremer in 2006 [68]. Ions with the same charge interact very differently with polyelectrolytes. For example, magnesium can only make special spatial configurations (octahedral) in electrostatic interactions with polyanions, while calcium has no spatial exigencies [66]. Katchalsky [6] cites a report of ionic interactions with polyacid alginate. The result showed that only 1% of calcium was free and 99% bound in complexes, while 15% of magnesium was free. One ion electrostatically immobilized had no detected activity. In the same study, 40% of sodium and 35% of potassium were free.

Ion concentration and activity are equivalent only in diluted solutions. At the synaptic space between membranes, a layer of 50 nm of polyelectrolytes creates enormous electric fields (300,000 V/cm^2^). The behavior of ions in this environment is simply different from their behavior in diluted solutions. Ionic activity can rise and fall according to ionization of the polyanions and their screening capacity to immobilize counterions.

High sodium solutions (120–140 mEq/L from the standard 100 mEq/L) on retinas had a negative effect on tissue excitability: the amplitude of the potential drop was to 40% of controls, its time derivative 23%, and propagation velocity 66%. The retina loses transparency in high sodium and the optical profiles become inverted: transparency increases when the potential drops [69]. Based on these results on retinal waves and the Hofmeister series position (Jones-Doyle viscosity* B* coefficient [67]) of lithium and sodium, we could predict pronounced lithium effects on excitable tissue. These effects have been reported [70–72]. Dietary lithium changed spread velocity of cortical spreading depressions. We have reports of negative effects on excitability in the single axon, as well as systemic effects in the heart and CNS. Therefore, we have an explanation for the general effect of lithium salts on mood swings. The probability of metastable states of the polyanions just changes decreasing excitability of axons, heart, and CNS.

### 4.1. The Extracellular Potassium Component of Circling Waves Experiments and Exogeneous Pulses

This type of self-sustained wave activity was created by Martins-Ferreira et al. in 1974 [23] (for its history, see [18]). The other type of self-sustained wave activity in retinas is the sequence of spiral waves [73].

A metal ring cut a circle of tissue in central retina, separating it from the rest of the tissue. A wave elicited on the outer ring had one wavefront killed by aspersion of magnesium chloride (4 mEq/L). The other front will keep circling for several hours (typically 3 to 4 hours). In most retinas, the inner circle remains quiescent but sometimes a region of it will start “spontaneous” (not deliberately elicited) waves. When the inner circle wave reaches the border, in the gap between the inner and outer circles, extracellular potassium activity increases. This gap is filled by the perfusion solution, set at 6 mEq/L of potassium (in this gap, concentration and activity are about the same). If the recording electrode in the outer circle position is at 0.5 mm or closer to the border, extracellular potassium activity transients are recorded associated with the inner circle wave. If the position is farther than 0.5 mm, no transient extracellular potassium appears in the recording. This observation shows the tissue long-range correlations for ion activities in the neuropil.

**Figure 3:**
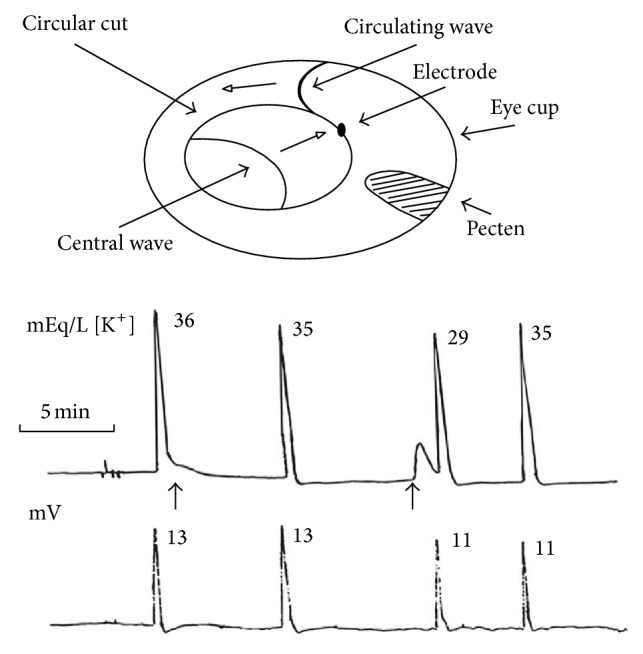
Schema of circling wave experiment with a potassium sensitive electrode inserted in the inner plexiform layer. A circular cut created an inner circular area separating the outer ring and the inner circle. The gap is filled with the perfusion solution. A wave is elicited in the outer ring and one wavefront is killed by aspersion with a high MgCl_2_ (4 mEq/L) solution. The perfusion rate is transiently increased such that the remaining wavefront is kept propagating. The electrode was positioned within 0.5 mm of the border of the circular cut. The potassium activity and the extracellular local potential are recorded simultaneously. At the side of each potassium activity wave and extracellular potential drop, the peak values are shown in mEq/L and mV. The values shown are typical of this type of experiment, as well as the shape of the potassium and potential wave concomitants. “Spontaneous” waves appeared at the inner circle and potassium activity increased in the solution gap and at the inner plexiform layer of the outer ring. Two potassium activity transients are shown (arrows). Note that both do not influence local extracellular potential, although the potassium activity waves were affected (expected in system with hysteresis). Note also that the relation between potential and potassium peaks is not linear (modified from [74]).

Figure 3 shows two extracellular potassium transients associated with inner circle “spontaneous” waves (arrows). The numbers on the side of the potassium and potential records show the peak value for each successive circling wave. These values are typical of spreading depression waves recorded in neuropil. The kinetics of both wave concomitants are of the more usual shape: an abrupt rise and long-lasting relaxation to prewave values.

The first inner circle wave alters the recovery of the extracellular potassium wave recorded in the outer circle. The second produces a potassium activity transient typical of exogenous potassium application. In the extracellular potential recording, there is no visible alteration caused by both extracellular potassium transients. Therefore, there is no simple relation between the kinetics of ion activity and extracellular potential recorded nearby (double barrel pipettes). Moreover, there is no linear rise in potassium preceding the abrupt rise that signals the wave onset, nor a potential rise within the neuropil associated with the second potassium transient.

What changes in the record of Figure 3 is the baseline potassium activity, which was 4 mEq/L at the end of the record. At the beginning, the value was 6 mEq/L, the same as the perfusion solution. The potassium baseline value at the end of the record is 4 mEq/L. This retina is maintaining extracellular potassium level below 6 mEq/L used in the perfusion solution for about 30 minutes. This is possible only if potassium is being pumped by glia and accumulated intracellularly or if the potassium activity is being checked by electrostatic screening.

The active transport of potassium is attributed to the Na/K ATPase. Reichenbach et al. [61] showed that the glial pump is driven by extracellular potassium and that its work is adaptive (the rate of transport adjusting to the potassium level). At 2 mEq/L or below, the pumping stops. At 12 mEq/L, it reaches the maximum rate. This maximum rate was estimated at billions of ATP cleavages (3 × 10^9^) per cell per minute. Glial Na/K ATPases can change the stoichiometric ratio of 3 Na for 2 K in situations of low Na, high K, or high depolarization. In these situations, potassium will be transported even in the absence of sodium. There must exist a synergic action between the kinetics of potassium channels and glial pumping rates; otherwise, the dissipation of potassium gradients would lead the glial cells toward potassium equilibrium in tens of seconds. We can see from the figure that the tissue maintains neuropil potassium activity not in equilibrium, with the perfusion solution at the inner limiting membrane being kept in this state for tens of minutes.

As shown in the figure, the interwave interval increases between the second and third wave and then decreases between the third and fourth ones. Usually, a retina maintains a uniform mean spread velocity for long periods (hours), if left undisturbed. The system can maintain the* metastable* state far from equilibrium for hours, with the circling wave still spreading during the relative refractory state. The shorter the absolute refractoriness, the higher the spread velocity in the relative refractory period (see [75, 76] for the details). Therefore, we can say that this retina had fluctuations on its absolute refractory* period* and was in a nonstationary state.

What did not change in the record shown in Figure 3 was the abrupt rise of potassium and the potential drop when the front wave invaded the region sampled by the electrode. In 74 circling waves, the peaks of the simultaneously measured time derivative of potassium activity and potential drop coincided [77], suggesting tight coupling between the two wave concomitants, although there is no linear causality between them. In a different series, the simultaneously recorded time derivative of potential and of the *µ*IOS (the brightness of a 50 *µ*m square pixel matrix overlaying the electrode tip inserted in the inner plexiform layer) of retinal waves also showed coincidence between the extracellular potential drop and the change in the optical signal [17]. In summary, the rate of fast changes at wave onset in potassium activity, potential drop, and optical properties are tightly coupled events.

**Figure 4:**
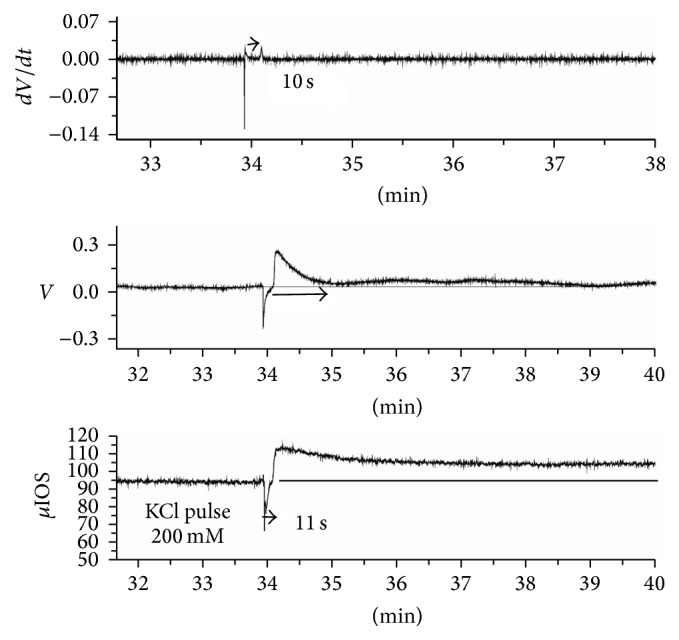
Experiment with fast high concentration KCl solution asperged over the inner limiting membrane (200 mEq/L and 200 *µ*L) with an Eppendorf pipette. The local potential and local IOS (the average brightness of a 50 *µ*m square matrix overlaying the electrode tip) were simultaneously recorded. In the upper row, the *dV*/*dt* time series, calculated from the potential time series, shows two separated peaks. The potential time series is in the middle row and the *µ*IOS in the lower row. Note that, in this context, both the IOS and potential show two components: a potential rise at the inner plexiform layer coincides with transient hypertransparency. We interpret this component as the “passive” response of the endfeet bilayer responding to the sudden potassium increase. Note that electroneutrality is broken for three seconds. Ten seconds later, the typical potential drop of retinal waves coincides with the typical wave light scatter increase. We interpret this response as the “active” response of the glial network/synapses, corresponding to the cooperative conformational change in the polyelectrolyte gel. The *µ*IOS time series recovery stops at a plateau level, as well as the potential time series, although the small negative potential only lasts 5 minutes. Note also that in the 10-second interval between the two components the system is at a supercritical state. Ten seconds separates the two *dV*/*dt* components and eleven seconds the two peaks of the *µ*IOS. The potential drop has the typical one-minute duration of retinal waves recorded at the inner plexiform layer.

The effects of exogenous potassium applied in retinas can be different from the ones shown in Figure 3 and reported previously for experiments with slow pulses [77]. If a very fast pulse of high concentration KCl (200 mEq/L and 200 *µ*L) is asperged with pipettes very close to the inner limiting membrane, then the local potential and optical response can behave as shown in Figure 4.

The simultaneously recorded local potential and the *µ*IOS time series were stored. Later, the time derivative was calculated [17]. Figure 4 shows the potential, the time derivative, and the *µ*IOS time series. Note that the extracellular local potential rises with pulse and with no latency measurable with our slow sampling rate (10 Hz). The potential rise recovers exponentially in about 3 seconds. Then, the system is at a supracritical state for 10 seconds, when the usual potential drop signals the onset of excitation propagation. The potential drop has the usual abrupt onset and slow relaxation in one minute, but the recovery is not complete: a small negative shift persists for five minutes. The time derivative shows two components clearly separated and in different directions.

The *µ*IOS shows an increase in tissue transparency with kinetics close to the potential rise and 10 seconds later the usual abrupt onset of light scatter at wave onset. The recovery of the tissue transparency stops at a plateau level (the duration of plateaus is 15–20 minutes in different retinas). The results show that the same agent elicits opposite electrical and optical responses, demonstrating that there is no simple relationship between ion activities and tissue transitions of states. However, “complex” does not mean “inscrutable,” and an electrochemical interpretation of these results is possible.

With fast pulses close to the inner limiting membrane, KCl is pushed toward the endfeet layer. In solution, potassium and chloride have the same mobility and they occupy the same relative position in the Hofmeister series. They are both named* chaotropic*. The rise in potential means that more potassium than chloride reaches the endfeet membrane. In other words, electroneutrality does not hold. The negative charges of the polyanions in the gel and the lipids heads of the bilayer will repel chloride, and the endfeet membrane potential rises just as it rises in lipid bilayers in response to fast solutions changes [78]. The endfeet membrane area is a considerable part of the total area of the glial cells, and the membrane is continuous with the rest of the cell, so that the whole surface cell membrane potential rises. The recording electrode inserted in the inner plexiform layer senses this potential.

There is a long held hypothesis that the Müller cells act as light guides. It is radial glia elongated from inner limiting membrane to outer limiting membrane, with its nuclei on the side of the body pushed out from the light pathway. Recently, experimental evidence appears to confirm this hypothesis (for a summary and relevant literature, see [45]). The endfeet shape acts as a lens and its refractive index matches the* humour vitreous*. The rise in surface potential of the endfeet changed the shape in favor of the light pathway through the retina, increasing transparency. This is one pathway of glial cells modulating retina activity, thus promoting functional plasticity.

The experiment shown in Figure 4 belongs to a series in which the macroscopic IOS also was recorded simultaneously with the time series displayed in the figure [17]. The hypertransparency was also recorded at macroscopic scale (1 mm diameter circular area of central retina). We estimated that about 15.000 Müller cells and 180.000 endfeet responded synchronously to the fast KCl pulse. It is possible that, inside the tubules of glia at the inner plexiform layer, the glial acidic protein filaments helped to create photonic crystals allowing more light to traverse the cell. After a few seconds, potential and transparency were back to baseline values; however, the excess potassium made the system unstable and a wave was triggered 10 seconds after the pulse. The potassium rise at the inner plexiform layer at the wave onset coincides with the second component of the *dV*/*dt* time series [77]. The potential drops and the tissue transparency falls abruptly. Again, a structural change in the glial network can explain the result: now conformation changes in the polyanionic gel liberate potassium from screening sites in a cooperative manner typical of “volume phase transitions” in these gels [3, 4]. The polyelectrolytes conformational changes change the membrane potential and ions activities. The loss of the structured interfacial water makes the hydronium ions associated with it disappear and pH shifts both intra- and extracellularly in the alkaline direction within the glial network [42, 43]. The loss of the photonic crystals decreases the tissue transparency (see section on deuterium).

### 4.2. The Extracellular Calcium Waves in Circling Waves Experiments and Intracellular Calcium Fluorescence in Solitary Circular Waves

**Figure 5:**
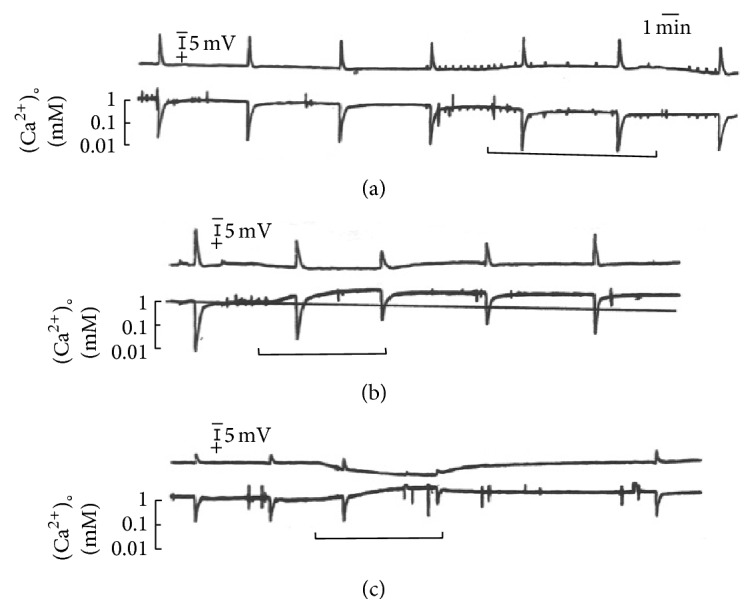
Circling wave experiment recording with calcium sensitive electrode and local potential glass electrodes inserted at the inner plexiform layer. (a) In the initial hour of the experiment, the circling wave spreads with uniform velocity. The extracellular calcium activity falls two log units from 1 to 0.01 mEq/L. The bar shows the change in the perfusion solution from 1 mEq/L to 0.1 mEq/L. Note that the fall in baseline calcium activity at the neuropil is very slow and reaches the value of 0.6 mEq/L at the end of the pulse. The wave transients do not change. Note the slower propagation velocity with low calcium. (b) 40 minutes later, the system returned to control values for the wave concomitants and propagation velocity. Flunarizin 2 *µ*M was applied for 8 minutes. The baseline calcium activity increased to 3.2 mEq/L at the end of the pulse. The wave concomitants were smaller. Note the increase in propagation velocity under the influence of flunarizin. It took 40 minutes of flunarizin washing off for the baseline calcium activity to return to control level. (c) Third hour of circling wave recording, one hour after the last wave in (b). Barium chloride (4 mEq/L) was applied. Note the depression of potential and calcium transient during turning waves. Barium was the agent which could depress the potential drop to zero and slow propagation to a factor of 5 times slower. There was an increase in the baseline calcium and a positive shift in the baseline extracellular potential in the presence of barium (figure modified from [79]).

The extracellular calcium activity was also recorded in circling waves experiments.  Figure 5 shows three segments in our experiment with duration of 4 hours.

In the initial period shown in Figure 5, the circling wave passes the region sampled by the calcium sensitive electrode at regular intervals and with the local potential drop and calcium activity fall with similar amplitudes in the successive waves. The tissue is in a steady metastable state. Then, the calcium concentration in the perfusion solution was switched from 1 to 0.1 mEq/L for 10 minutes. For 3 minutes after the solution was equilibrated in the retinal chamber, calcium activity in the neuropil did not change; after that, it begins a slow fall reaching 0.6 mEq/L at the end of low calcium perfusion. The baseline calcium activity was back to control 10 minutes after the end of the low calcium perfusion. There was a small and slow negative shift of the local potential. The amplitudes of the electrochemical wave concomitants did not change but the recovery of the calcium transient was somewhat extended. It seems that immobilized calcium within the gel was being liberated in order to maintain homeostasis. Ionic exchanges at the membrane surface can explain the results.

The abrupt fall in calcium activity at wave onset begins at the peak of the local potential drop and potassium increase derivatives. The fall in activity is one to two log units in different retinas. The recovery to baseline apparently has a fast and a slow component. Waves with only a small amplitude and slow kinetics calcium activity have been occasionally recorded (see [80]). However, more frequently, we recorded circling waves without changes in calcium activity (see the next section). Calcium differs from the monovalent cations and magnesium in the ability to make cross-links provoking conformational changes in polyelectrolytes (as we already commented in the results with alginic acids measurements of 99% bound calcium). All membrane receptors and channels are glycoproteins with the sugars shown as tree branches protruding into the extracellular gel. It is possible then that most of the fall in activity is due to the ion-polymer interaction and not to the transfer through channels from the extra- to the intracellular compartment. Calcium activity increases intracellularly in astrocytes at wave onset, but not always [43, 81]. Measurements with calcium sensitive dyes in* in vitro* retinas showed that even in one spreading wave calcium signal could be absent in large patches of the tissue; in [82], the scatter of red light and of exogenous calcium dye fluorescence is shown, in which a large patch of a spreading wave lacks both signals, while the scatter of green light was seen in the area. In [82, 83], it was shown that barium addition in the perfusion solution blocked the intracellular calcium signal and the red light scatter of a propagating retinal wave, without affecting the green light scatter of the wave.

Barium was also applied to circling waves.  Figure 5(c) shows an example of barium application to a circling wave experiment. The bar shows the time when the barium added solution (4 mEq/L) reached equilibrium. The local potential rises slowly and the potential drop associated with the waves becomes smaller and smaller. The baseline activity of the calcium electrode shows an increase and a smaller calcium transient in the second wave recorded in the presence of barium. Barium also had profound effects on the kinetics of the extracellular potassium wave transients (see [80]). The peak amplitude was increased and the recovery slowed down. Also baseline potassium was maintained below 6 mEq/L for a prolonged period. However, the more pronounced effect of barium was in the spread velocity of circling waves. An effect on the absolute refractory period could explain the slower spread velocity of circling waves.

Barium does increase the absolute refractory period; it almost doubles it [76]. In two separate sets of mechanically elicited, solitary waves experiments [69, 83], barium had a marked effect on the amplitude of the local potential drop and the spread velocity of retinal waves. In one of the experimental series [69], from 10 out of the 17 waves elicited in the presence of barium, 7 had complete depletion of the potential amplitude. The other 10 waves appeared in (a mean of) 39% of controls with the peak of the derivative appearing in 31% of controls. However, barium did not change the shape of the dispersion relation curve but only shifted it to lower values for all the interwave intervals tested [76]. The effect on the spread velocity thus appears to be independent of metabolic effects of barium. Physicochemical or lyotropic effects appear to dominate barium effects. For monovalent cations, the position in the Hofmeister series correlates well with the value of the Jones-Doyle viscosity* B* coefficient [67]. This coefficient is a direct measure of the ion-water interactions normalized for water-water interactions. The* B* coefficients for cations are magnesium 0.385, calcium 0.285, barium 0.220, lithium 0.150, and sodium 0.086. They are all said to be* kosmotropes*. Potassium −0.007 and cesium −0.045 are said to be* chaotropes*. For example, calcium is prone to promote aggregation of macromolecules (or even membranes), one of the mechanisms behind “volume phase transitions” of polyanions. These effects are apparently used in nature to form tight junctions and desmosomes. Lithium has 14 times the affinity for carboxylate side chains in peptides compared to magnesium [84]. Magnesium has spatial constraints for charge interaction [66].

Figure 5(b) shows the marked effect of flunarizine [79] on the baseline activity of calcium in the neuropil. It reached 3.2 mEq/L and persisted for more than 20 minutes after the pulse. After forty minutes, the baseline calcium was back to 1 mEq/L. Unlike barium, flunarizine increased the spread velocity of circling waves (*n* = 4 retinas and 6 pulses). Low calcium perfusion slows down propagation, but there is no simple relationship between high calcium activity and spread velocity in circling waves experiments.

A detailed study on the role of extracellular calcium and propagating retinal waves used zwitterionic (phosphatidylcholine, PC) and negatively charged phospholipids (phosphatidylserine), as well as sialic acid, to compare their effects with exogenous applied gangliosides [85]. Sialic acid (1 mM) was able to make the tissue unexcitable in about 2.5 hours. Its effect was washed off in 30 minutes. Gangliosides had a similar effect at 50 *µ*M. The more glycosylated the side chains, the more pronounced the effect. Washing the tissue for two or more hours did not reverse the ganglioside effect. Spread velocity and the shape and intensity of the IOS also were depressed by sialic acid and gangliosides, either in solitary circular waves protocols or in circling waves. PC had no effect and PS smaller effects than gangliosides at 500 *µ*M. EGTA at 800 *µ*M had smaller effects than the gangliosides (at 50 *µ*M).

Gangliosides are found exclusively at the outer leaflet of lipid bilayers of mammalian membranes. They form the nearest polyanion layer to the lipids and trap calcium ions. The depression of the IOS amplitude by gangliosides suggests that conformational changes at the endfeet membrane were responsible for the optical and spread velocity effect on the waves.

### 4.3. Wave Propagation with Nonstationary Glial-Neural Dynamics in the Neuropil

Circling waves experiments are especially suited to observe the conditions compatible with the spread of excitation waves within the neuropil. The absence of the local potential drop and of the calcium activity fall transients was the common absence recorded. Extracellular potassium increase in activity was always present but its shape and peak values could vary.

**Figure 6:**
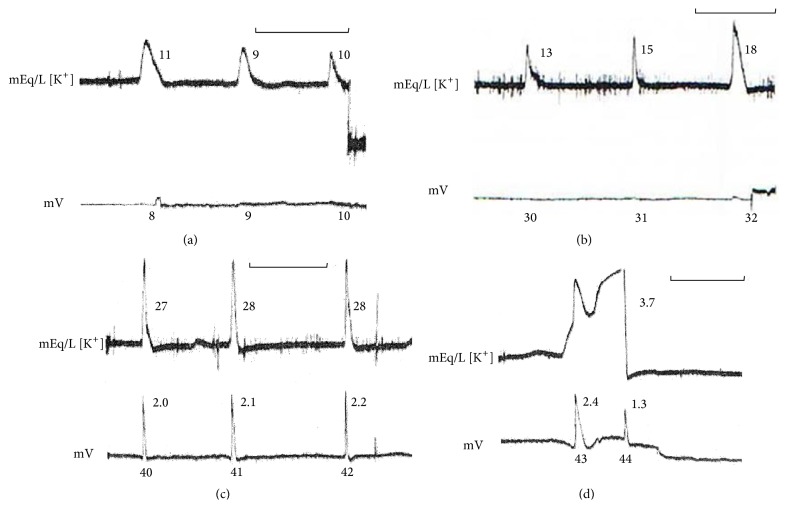
Plastic dynamics between the glia network and synaptic terminals in a circling wave experiment with 4-hour recording time. Potassium sensitive electrode and local potential recorded at the inner plexiform layer. The horizontal bars in each square show 7 minutes of time in the record. Below each wave is the number of the turns that reached the electrode tip. In the first 31 turns, the local potential did not show the “typical” potential drop and the extracellular potassium activity wave change showed low amplitude and changed shape (or kinetics). Then, at turn 32, a small potential drop appeared and the peak of the potassium waves increased to typical values. The potential waves also increased in amplitude but remained one order of magnitude smaller than the “typical” values (above 10 mV). Note the fast kinetics of the potential and ion activity waves at 40–42. Note also the small oscillations of potassium activity in (a) and (c) before the long (order of minutes) potassium increase that interacted with two propagating neuropil waves. The long potassium activity transient begins with a linear growth and potential rise within the neuropil; then, a second linear growth interacts with the turning wave invading the area and the extracellular potential drops. This second linear growth also had an IOS that showed a standing pattern that lasted for 24 minutes and faded without leaving lesions behind. The value of potassium activity reached 37 mEq/L. This value was maintained for 24 minutes and the return to baseline control levels took another 20 minutes. As usual, one wave was elicited at the border of the standing pattern and this wave killed the previous circling wave on collision. One hour after the circling stopped, the retina was readily excitable by mechanical touches and 8 waves were elicited with shape and amplitudes similar to the ones in 40–43 turns. This experiment illustrates well the plastic dynamics between glia and synaptic terminals membranes interactions and the nonlinear system response to any particular macroscopic wave concomitant precluding linear causality among these concomitants (modified from [79]).

Figure 6 shows one circling wave experiment in which for 31 turns of the circling wave the local potential drop did not occur; then, from wave 32 to wave 42, a small amplitude potential drop appeared (around 2 mV). The circling stopped when a marked increase in extracellular potassium activity elicited a “spontaneous” wave that collided with the original circling one, annihilating each other. Two waves were recorded by the electrode within this potassium activity transient in which the activity reached 37 mEq/L.

The extracellular potassium transients had a low amplitude and variable shape in the first 10 turns and began to stabilize in the typical shape and grow in amplitude at turn 32. Note the two small oscillations in the baseline potassium activity, the second just before the prolonged high amplitude transient. The observed IOS of the circling waves ensures the existence of propagation.

**Figure 7:**
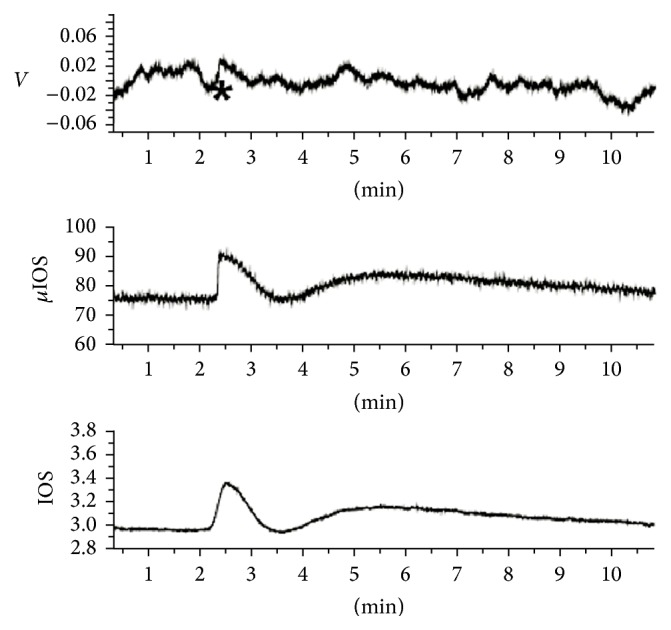
Extracellular potential recorded at the inner plexiform layer and the wave IOS recorded simultaneously. The IOS was recorded at two different spatial scales. *µ*IOS time series depicts the mean brightness of the 50 *µ*m square pixel matrix overlaying the electrode tip and IOS the photomultiplier output that samples the scattered photons of a circular area of central retina with a 1 mm diameter. The wave recorded was from a series of 44 isolated circular waves from 34 retinas [17]. This wave had a “zero potential” record at the usual amplification. However, when the time derivative of the *µ*IOS was calculated, a small sharp transient (about 200 *µ*V/100 ms) coincided with the peak of *dµ*IOS/*dt*, suggesting that asynchronous small transients were present at the inner plexiform layer instead of the typical synchronous potential drop at wave onset. Note the sharp onset of the *µ*IOS, whose peak coincided with the macroscopic IOS peak (the electrode tip is at the centre of the photomultiplier's sampled circle) and slow smooth growth of the IOS time series: the photomultiplier is integrating (summing up) many *µ*IOS signals from a large area.

Figure 7 shows a record of a solitary circular wave with zero potential drop. In this series, local potentials were recorded with the IOS at two spatial scales: 1 mm circular area sampled by a photomultiplier (labelled IOS in the figure) and the pixel brightness of a square matrix overlying the region of the inserted electrode tip calculated from digitized frames from a camera (labelled in the figure as *µ*IOS). The local extracellular potential (labelled* V*) is the upper row time series. The three time series were simultaneously recorded. Note the abrupt rise of the IOS at the micro spatial scale and the smooth growth of the photomultiplier signal. The photomultiplier is integrating the light scatter of all *µ*IOS within the circular area, such that the peak of the photomultiplier signal is set at the point when the wave covers half of the sampled circular area. The peak of time derivative of the *µ*IOS and of the local potential matched in 44 circular waves of 34 retinas [17]. Therefore, in this wave, we found the peak of the time derivative of the *µ*IOS and looked at the local potential time series to see what was happening. A very small (order of *µ*V), sharp transient coincided with the maximum rate of change of the *µ*IOS shown in the figure (marked by an asterisk). If the optical properties of the tissue depend to a large extent on the light guide role of glial cells, then this geometric change can happen and propagate without the local* synchronous* potential drop in the neuropil of the inner plexiform layer.

**Figure 8:**
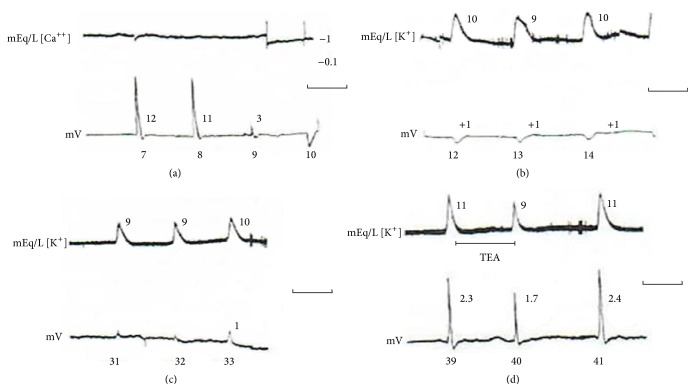
Circling experiment with 4-hour recording time and changing extracellular potential and ion activity concomitants. The horizontal bars show the recording length of 7 minutes. (a) First-hour recording time, waves 7–10, calcium sensitive electrode inserted at the inner plexiform layer. Only wave turn 7 has a small extracellular calcium transient. The extracellular potential drop looked typical for turns 7 and 8 and dropped markedly in amplitude for turn 9. The electrode was reinserted nearby (about 1 mm away) and the calcium transient was still zero with the extracellular potential changing direction from negative to positive for turns 10 to 20. (b) Second-hour recording time; the calcium electrode was replaced by a potassium sensitive electrode. Wave turns at 12–14. Low amplitude and variable shape of the potassium activity wave and potential rises in the extracellular inner plexiform layer occur. The “positive” potential waves were present from turns 10 to 20, and from 21 to 26 there were “zero potential” waves. (c) Third-hour recording time. Small potential drops reappear and persist from turns 33 to 39, when a pulse of 3 mM TEA was applied. TEA depressed the amplitude of potassium and potential waves and slowed down their propagation velocity (modified from [74]).

Figure 8 shows a circling wave experiment in which for 10 turns the ion-sensitive electrode did not show calcium transients. The local potential had the typical shape and amplitude and then lost amplitude, inverted to local potential rises in the next 20 turns, and inverted to potential drops again for the next 11 turns. Other examples of “positive potential” waves are shown in [80]. At turn 11, the recording electrode was changed to potassium sensitive and the extracellular potassium transients associated with the circling wave show variable kinetics and low amplitude until the end of the recording. These experiments show clearly the nonlinearities in the system. Just as there are several B-Z system “recipes,” excitation in the neuropil propagates with the interacting glial-synaptic membranes at different states and these states can change within a circling wave experiment with 3 to 4 hours of recording time. In other words, it is a flexible dynamical system.

## 5. Conceptual, Physical, and Mathematical Models

In this section, we apply some concepts derived from chemical and electrochemical excitable media to the brain. Some predictions derived from the field and from the physical-chemical properties of polyelectrolytes have been fulfilled and they help to understand the brain dynamics.

What is now termed the field of excitable media had, at its origin, the confluence of three factors: the fast increase in computer power for simulations, the discovery (by western researchers) of the chemical system created by Boris Belouzov in the fifties of the XX century [19, 86], and, almost simultaneously, the acceptance of the theoretical model of Ilya Prigogine for chemical self-organization [21]. The recognition of the spreading depression wave as a phenomenon akin to the B-Z system by one of the leaders of the spreading depression research, Bureš, led to the second prediction about these CNS waves: the existence of evolving sequences of spiral waves [73, 87].

Forty years later, the existing knowledge about these systems permits some generalizations. First, the system where they occur must be far from thermodynamical equilibrium (plenty of energy is available for dissipation); second, positive feedback of some kind is present at the wave onset (autocatalysis in chemical systems, interfacial electrical field at electrochemical systems [88]). There is slow inhibition following the explosive growth of a component of the system.

Mathematically, a nonlinear (quadratic or cubic) growth, followed by linear slow decay, is the minimum requirement for self-organization. These systems exhibit a rich behavioral repertoire. The system has at least three states: quiescent but ready to be excited, excited, and refractory to excitation. The refractoriness goes from absolute to relative before the system is back to quiescence. For example, both computer simulations and experiments with bulk reactors showed that the temporal behavior of the B-Z system changed from a simple limit circle to complex periodic states to chaotic behavior, depending on the rate of pumping matter or chemical energy in the system. In two dimensions, waves appear in the form of either target patterns (Figure 1) or sequence of spirals [73].

The heart can be conceived as a machine designed to produce excitation waves in three dimensions, while the axons of neurons produce them in one dimension. This knowledge explains why the retina is such a good model for experiments with two-dimensional excitation waves: the CNS slice has an intact network in which only the output axons are cut behind the sclera. In the perfusion solution, glucose concentration (30 mM) is higher than the physiological 20 mM found in the avian liquor. Furthermore, the network is wired for transduction of electromagnetic energy; in order to observe the waves, illumination is used. This second source of energy in retinas is physiological. Only good shape retinas with plenty of energy to expend will display waves. The fact that the initial phase of the wave is very similar to the onset of excitotoxic responses brings about some confusion for experiments with other preparations. The complete optical profile of these two states makes them easily recognizable in retinas [44] and the recovery of excitability after an episode of excitotoxic response ensures the functionality of the network.

Both cortical and retinal neuropils display excitation waves, but with different probabilities and geometry of the wavefront, due to the differences in the geometry of the glial network [18]. The same structure will also display macroscopic oscillations (alpha and theta EEG cortical rhythms) and standing patterns (a good guess for* petit mal* seizures).

Katchalsky predicted that electrochemical patterns in the brain would happen at the suprasynaptic or macroscopic scale and that wave propagation would show coherent flow of matter or energy. An almost horizontal dipole standing around a mechanical stimulus zone (see Figure 19 of [83]) produces a pattern that shimmers and shifts in place, suggesting a dynamic pattern of space/time energy oscillations. The predicted coherent flow was demonstrated by Rovinsky and Menzinger [89] in a series of experiments with the B-Z system. They constructed an almost one-dimensional system, but one can also say that they created a system analogous to the neuropil: they filled a tube of 3.2 mm diameter and 25 cm length with 40 *µ*m microspheres coated with cation exchanger resin (sulfate polyanion). Upon this coat, ferroin was loaded to act as catalyzer. The microspheres had 40 *µ*m average diameter and they were packed to fill the maximum packing density, leaving 30% of the tube volume for the reactors solution. In other words, a convoluted space with a high surface-to-volume ratio separates facing reactive surfaces, in a similar fashion to the neuropil. Coherent flow was demonstrated beyond any doubt. The propagating wavelengths were in the order of centimeters. The authors called attention to the presence of the same organizing principle in electrolytes in the presence of energy fields, as predicted by Katchalsky.

Another significant result from excitable media is the almost perfect two-dimensional system of Flätgen et al. [88]. The authors recorded images of potentials drops and two-dimensional waves propagated at velocities of meters/second, the order of magnitude of action potential propagation. They stated that they did not interpret this as mere coincidence, and neither do we. If Tasaki's membrane model is used, then the action potential is a surface or interfacial electrochemical phenomenon: “The cortical gel layer of nerve fibers has the properties of a cation exchanger. Hence, this layer can, and actually does, undergo a reversible abrupt structural change when monovalent cations (e.g., Na^+^) are substituted for the divalent counter-ions (e.g., Ca^2+^). This structural change brings about a sudden rise in the water content of the layer, which in turn produces a large enhancement of cation mobilities accompanied by a shift of ion-selectivity in favor of hydrophilic cations” [4]. In the present interpretation, interfacial water also transiently changes states from the structured interfacial water to bulk liquid.

However, the most interesting model of excitable system, in the present context, was created by Wüssling and collaborators in 1999 [90–92]. They inserted fragments of sarcoplasmic reticulum membrane of muscle in the polyanionic gel of agarose and demonstrated optically the existence of calcium activity waves. All the requirements of excitable media were present (the first of their papers [90] is a good introduction to the field). Furthermore, these experiments show how the experimental context changes the behavior of the system: the addition of mitochondria to the gel and membrane increased the wave propagation velocity in the first publication; however, changing the distribution of the elements from aggregates to a more uniform one changed the response to mitochondrial activity. This excitable medium is probably the simplest model for biological relevant ion activity waves, and its constituents are a polyanionic gel with positive feedback on calcium release by ryanodine receptors. The slow kinetics of the transporters provide the negative feedback. Ryanodine receptors have been identified as a source of cytoplasmic calcium on astrocytes [93].

Katchalsky also predicted that what he called the mechanoelectric coupling of polyelectrolytes could be used in biology when phase transitions would produce mechanical work. This is exactly the case for the mechanical behavior of inner ear hair cells: the integral membrane protein prestin has a prominent intracellular loop and is expressed in assemblies of molecules. A change of monovalent anion to a divalent one at the intracellular loop triggers the cooperative conformational change (phase transition) that spreads through the assembly [94, 95].

The biological significance of the results with Nafion gels can be found by means of a description of exclusion zones (EZs) in microvessels. Venules and capillaries have 0.5 *µ*m zone that excludes red blood cells and colloidal particles [96–98]. The structured water around the polyelectrolyte fixes the negative charges vibrating in place. The important points here reiterated are as follows: the structured water is a very dynamic and fragile structure that promotes charge separation, and charge separation is energy. The vessels use this energy to facilitate the flow. Nafion tubes submerged in water showed self-driven flow in the absence of pressure gradient [99]; furthermore, white and UV light increased the flow velocity.

## 6. Discussion

We have assumed an excitable media and polyelectrolytic approach to explain ionic oscillations and waves in neuropil. We also interpreted B-Z with deuterium experiments from an electrochemical point of view. Usually, B-Z is seen as a chemical self-organized system. However, the role of hydronium ions in organizing coherent flow is the simplest explanation for the excitability collapse of B-Z systems and retinas. There is little doubt now that water can store charge [35, 100, 101]. Therefore, interfacial water is not electroneutral, including the unstirred layer of bilayers and the hydration of polyanions of the extracellular and intracellular gels.

The structure of the neuropil membranes and interacting gel between them generates electrical fields; fields imply forces, and forces can direct a coherent flow of charges (e.g., in hydronium ions). A direct demonstration of this type of far-from-equilibrium flow has been published [99]. This effect was not fully taken into account [89]; although a residual flow due to gravitational field was recognized, the additional electrical field effect was not considered in the interpretation of the behavior of excitable media. The polyanionic gel was considered to be just a passive element [90–92], since the deuterium effects on retinas and B-Z were first presented [28, 29]. With hindsight, the intrinsic electrical field effects could be the unifying factor in all these excitable media.

Bunkin studied the optical properties of interfacial water and demonstrated its birefringence [37, 38]. It is long known as the birefringence of axons (e.g., [1]). He called attention to the similarity of the optical properties of interfacial water and quasi-liquid crystals [39] (photonic crystals behavior of this water). The first photonic crystals described were dielectrics. The pioneers of proposing the importance of the dielectric properties of brain and cells were Frölich and Adey [102–106]. The intrinsic electric field of the neuropil membranes and the polyelectrolytes between them compose a vibrating, self-organizing system. Macroscopic patterns can be formed, as we have seen with a large dipole (Figure 19 of [83]). These patterns can store information, as proposed by Neumann and Katchalsky [11]. The brain, heart, and kidney could be systems for which the state of water is functional.

The proposal of Hameroff and Penrose [107] of using quantum mechanical concepts to explain consciousness has one direct piece of experimental evidence: the resonant vibrations of energy found in a single* wet* microtubule (then promoted to a nanotubule status) [108]. However,* dry* microtubules did not show this energy. The authors probably were not aware of the work of Pollack and Bunkin showing that water interaction with microtubules is far from unique. The Nafion experiments and exclusion zones of microvessels suggest that the energy of interfacial water is ubiquitous in physiology. The brain, after all, could be a “smart gel” machine [109] in which some phase transitions are perceived as functional pathologies (epilepsies, migraine aura, and transient global amnesia).

The use of concepts from nonlinear thermodynamics has the additional advantage of permitting experiments at very convenient time and space scales, without the abstraction of the concepts derived from quantum mechanics, such as the famous “wave collapse” that has been the object of a long controversy among specialists. Focusing on these spatiotemporal scales does not imply complete abandonment of quantum-like concepts, since an understanding of the properties of ions and their biological milieu, as well as their interactions, demands explanations based on quantum theories.

The distortion in perception described in migraine aura, hallucinations in the onset of complex seizures, and the impairment of learning and memory described by experimenters with cortical spreading depressions show how electrochemical patterns are close to higher mental functions [8, 110, 111].

A question able to suggest further experiments concerns the little discussed aspect of the asymmetry of the biological membranes lipid bilayers and associated gels. The zwitterionic phosphatidyl choline predominates in the external leaflet of human red cells membranes, while the negatively charged phosphatidyl serine predominates at the internal leaflet. Computer simulations showed that this asymmetry creates electrical fields [112]. Furthermore, glycolipids (gangliosides) and glycoproteins are exclusive of the external leaflet. The polyanion of the associated gel has carboxyl and sulfate as the predominant fixed anions in the external face, while phosphates are found in the intracellular milieu. Volume phase transitions [113] appear to be the conformational change that spreads at the external gel, while polymerization is the main change in the intracellular gel. The significance of this asymmetry alone could sustain a research program. We have measured in the retina long-range correlations of the order of 300–500 *µ*m (see Figure 3 in this paper and [114]). Another experimental result that is still in need of explanation is the fact that the light scatter of the retinal IOS has a red component that differs from the green light scatter in temporal and spatial distribution [83, 114–116]. The red scatter follows closely the potential drops and preceded propagation, displaying fluctuations, while the green scatter is the hallmark of propagation. Barium blocks the red scatter and glycerol predominantly the green part of the IOS. The second component of optical profiles has close coupling to metabolic acceleration [117, 118] and follows an acid shift in the extracellular compartment of the neuropil [42]. The kinetics of this component are independent of spatial scale [17] and its IOS spectrum has broad smooth appearance [115]. The green red preference found in the first component of the optical profile experimental manipulations is another issue for further investigation.

## 7. Concluding Remarks

Early interpretations of the function of astroglial ionic waves concentrated on neuroastroglial bidirectional glutamatergic mechanisms activating calcium waves. This interpretation was recently put into question by experiments with IP3 pathway knockout mice, revealing that blocking the release of calcium ions from the endoplasmatic reticulum mediated by the inositol pathway does not cause serious behavioral impairment in cognitive tasks [119]. While glutamatergic gliotransmission was questioned, new findings have suggested important roles for the astroglial control of potassium homeostasis in brain function [120, 121]. The findings reviewed in this paper suggest an original interpretive hypothesis addressing the mechanisms of neuron-glia interactions and related cognitive functions: the movements of calcium and potassium ions inside astrocytes and from astrocytes to neurons compose* a coherent hydroionic wave* with essential roles in brain and mental functions [122–124].

The activity of extracellular potassium and calcium changes the structure and state of excitable membranes in both glia and neurons. The kinetics of the sodium pump as well as membrane channels are changed in both networks with consequent changes in the intracellular ionic environment and metabolism. The spread of such changes is continuous in brain tissue and depends on the integrity of extracellular polyanionic gel and its capacity for phase transitions. This capacity is not impaired in IP3R2 knocked out mice [119], since Ca^2+^ signaling in astrocytes relevant for the hydroionic wave and related phase transitions is dependent on the ryanodine.
